# Annual Meeting of the New York State Medical Society, February, 1849

**Published:** 1849-04

**Authors:** 


*Annual Meeting of the New York State Medical Society, February,* 1849.—As it will be several weeks before the Transactions of the State Medical Society for the present year will be issued, we copy the following brief notice of it, with the list of officers, from the Albany Evening Journal:—

At the recent Annual Meeting of this Society, held in this city, it was gratifying to observe the unusually large attendance of medical gentlemen from different and remote parts of the State.

It is an evidence of the increasing interest felt by the medical public in its proceedings. The very able and interesting address, delivered by its President, Dr. ALEXANDER H. STEVENS, in the Assembly Chamber, before the members of the Society, members of the Legislature and citizens, gave an additional interest to the meeting.

The subject of the address—Medical Education, would at all times commend itself to public attention. But the masterly ability with which the subject was presented by the distinguished author, gave it new interest, and wrought conviction in the minds of all, of the necessity of granting every facility for the attainment of a thorough medical education, to those who shall hereafter enter the medical profession.

The following officers were elected for the ensuing year:

President—ALEXANDER H. STEVENS, M. D.

Vice President—ALEXANDER THOMPSON, M. D.

Secretary—P. VAN BUREN, M. D.

Treasurer—P. VAN O'LINDA, M. D,

Censors—*Southern District.*—Drs. J. R. Manly, John Cheeseman, Chas. S. I. Goodrich.

*Eastern District.*—Drs. Joel A. Wing, Thos. B. Blatchford, T. R. Beck.

*Middle District.*—Drs. Augustus Willard, Jenks S. Sprague, John McCall.

*Western District.*—Drs. Bryant Burwell, William Taylor, John Coats.

*Committee of Correspondence.*—Drs. Alonzo Clark, Barlow Wright, John H. Wheeler, Hiram Corliss, Helon F. Noyes, Augustus Willard, Phineas H. Burdick, Bryant Burwell.

The following gentlemen were elected permanent members: Drs. Alexander H. Stevens, New York; John M. Pruyn, Columbia; Morgan Snyder, Montgomery; Patrick H. Hard, Oswego; Dyer Loomis, Chenango; Enos Barnes, Ontario.

The following gentlemen were elected honorary members:—Alonzo Clark, M. D., New York; Joseph Parish, M. D., Philadelphia.

The following were elected delegates to the National Medical Convention:

*Southern—Censorial District*—Drs. A. H. Stevens, New York; Charles S. J. Goodrich, Brooklyn; Willard Parker, E. G. Ludlow, J. R. Manly, New York.

*Eastern District*—Drs. R. G. Frary, Columbia; Simeon Snow, Montgomery; J. McNaughton, Albany; T. W. Blatchford, Rensselaer; T. R. Beck, Albany.

*Middle District*—Drs. Henry Mitchell, Chenango; Dehring, Utica; J. McCall, Utica; Jenks S. Sprague, Otsego; A. Willard, Chenango.

*Western District*—Drs. Reynold Coats, Ontario; Daniel T. Jones, Onondaga; Alexander Thompson, Cayuga; Bryant Burwell, Erie; G. W. Bradford, Cortland.

*Transactions of the Medical Society of the State of New York.* Vol. vii. Part 3.

Since the foregoing article was in type, a copy of the published transactions of the State Society has been received. In addition to the address by Dr. Stevens, it contains a paper *On the Diseases of Onondaga County. By D. T. Jones, M. D., of Baldwinsville, Onondaga County.* It is a short communication, and will probably be found in the Eclectic Department of the next number of this Journal.

In glancing over the proceedings, the only interesting matter which arrests our attention, is the recommendation of the Society to the County Societies, to provide in their By-Laws for the constitution of a *Primary Board*, for the examination of young men about to commence the study of medicine, and requiring that the applicants shall, in addition to a good English, academic education, have made respectable attainments in the Latin and Greek languages, and that he shall sustain a good moral character. Of a committee of five who reported this plan, two were from this city; Dr. F. H. Hamilton, chairman, and Dr. B. Burwell. The society in this county, anticipated the action of the state society, by the adoption of the same measure at its annual meeting in January last.

Dr. B. Burwell, of this city, is one of the delegates to the National Association appointed by the State Society.

